# Supplementary material for: Analyzing a sport for development program’s logic model by using key actors’ perceptions: The case of Pour 3 Points organization in Montreal
Source: PLoS One. 2022 May 12;17(5):e0267785. doi: 10.1371/journal.pone.0267785 (PMC9098001; doi:10.1371/journal.pone.0267785)
Supplement: S1 Appendix — (DOCX) [file pone.0267785.s001.docx]

**S1 APPENDIX – P3P PROGRAM’s TRAINING**

***Context and recruitment***

Pour 3 points (P3P) is a non-profit SFD organization established in 2013 in Montreal, Canada, that used sport as a tool to foster youth development in low socioeconomic neighborhoods.

At the time of the study, the organization provided a two-year coaching training program for young Canadian adults who were interested in coaching and were willing to take on the long-term engagement of the program. During their enrolment in training program, they obtained skills to become life coaches while coaching sports at one of the organization’s partner schools.

Each year, the program recruited approximately 15 coaches based on background and individual interviews.

***Structure of the program for 1 year***

| Retreat | 4 days outside of the city |
| --- | --- |
| Peer discussion circles | 5 per year  Under the supervision of a mentor discussion of issues of the practice on the field |
| Formal trainings sessions | 5 per year  Conferences on specific thematic |
| Personal evaluations each year | 3 per year |
| P3P events | Around 7 per year (e.g., for funders, partners, organisation) |

P3P programme is based on sport coaching literature, including the Positive Youth Development in Sport approach and its 4C’s outcomes (Competence, Confidence, Connection and Character), and the types of coaching knowledge (i.e., intrapersonal, interpersonal, professional, and environmental). Many experts contributed to the development of the program.

**More information about the program is available here:**

<https://pour3points.ca/en/le-programme-de-certification-en-coaching-pour-3-points/>

<https://pour3points.ca/en/become-a-coach/>
