# Supplementary material for: Analyzing a sport for development program’s logic model by using key actors’ perceptions: The case of Pour 3 Points organization in Montreal
Source: PLoS One. 2022 May 12;17(5):e0267785. doi: 10.1371/journal.pone.0267785 (PMC9098001; doi:10.1371/journal.pone.0267785)
Supplement: Attachment — Submitted filename: Evaluation PlosOne P3P round 1 TG VF 19 11 2021.docx [file pone.0267785.s002.docx]

**PONE-D-21-03672**

**Analyzing a SDP program’s logic model with key actors’ perceptions The case of Pour 3 Points organization in Montreal.**

**PLOS ONE**

**REVIEWER 1**

| **COMMENTS** | **RESPONSES TO REVIEWERS** |
| --- | --- |
| Reviewer #1: Firstly, congratulations to the authors on a worthy piece of research focussing on often overlooked process of evaluation with SfD. See below for some specific and general comments. | Thanks for your kind comments. |
| Line 63 – Please clarify what you mean by acclaimed programmes and why this is negative. This term on its own does not clearly portray the issue within the cited paper. (11) | Please note that Levermore stated these 3 limits in his review of the literature concerning SDP, we just referenced their study. The author referred to the idea that only well know programs of SDP are evaluated through a rigorous process of evaluation because organisation have resources to do so. We now made a few modifications in the text concerning this point (l.64-65). |
| Line 116 – More detail required on what P3P actually delivers and what their implementation looks like in terms of the types of training they deliver, how this training is used within schools, does it have a timescale, is it only a training programme or does it provide more structure within the schools? I was still unclear by end of paper what the P3P model looked like, and it is discussed in very general terms. Example trainings or activities would be beneficial (can be included within Appendix if word count is an issue). | Details of the program are mentioned on page l.140-143 (Each year, the program recruited approximately 15 coaches. Throughout their enrolment in the program, the coaches participated in a four-day training retreat, five peer discussion circles, five formal trainings sessions, and three personal evaluations each year). We added Appendix A and the organisation’s website site, which describes the present program and its schedule. |
| Line 181 – What kind of coding utilised? | Coding was conducted following deductive (preestablish with elements of the logical model) and inductive processes based on Paillé & Mucchielli (2021) conceptual categories. After reading the verbatim, content was placed into groups of ideas and subgroups. We made modifications in the text concerning this point L.234-239. |
| Line -209 – Touched upon here but would like to see more detail around reflexive practices carried out among the research team. Giving an example of bracketing process used may help. | This comment is relevant, and we now provide details on this aspect. Modifications were made in the text (l.227-231). We hope that our response address’ your comment. |
| Line 214 – More detail around field notes, what they consisted of and their purpose within rigour of study would be beneficial. | We added some details and a reference about the field notes, we hope the rigour is better now. (l.232) |
| Line 228 – Personally I would like the theme school problems to be a bit more descriptive, when I hear the term I think of problems with the school structurally whereas the description breakdown looks more at personal challenges faced by young people at school. | Thanks for this comment. We now give details about it (see table 1). |
| Line 465 – o to or typo | We made the change. |
| General Comments  The aims of the study focus on assessment of a logic model however there are many parts of discussion (and abstract) lean into impact. The study is not designed to explore effectiveness of associated impact, nor would this be an appropriate study design to do so, however some claims ‘Positive changes with good potential to be sustained in the longterm’ do make such claims. Please review language around such comments noting these are perceptions of involved stakeholders. Later mention of need to continue work with participants, parents etc does acknowledge this but review language to avoid confusion. | Thanks for your comments. You perfectly understand what the aim of this study is and what we intend to do.  Following your comments, we decided to elaborate more on the impact of our study in the discussion and the abstract.  Also, we change some inappropriate claims along the text about exploring the effectiveness of the associated impacts. |
| Secondly, I agree with your recommendations around the need for clarification around multiple aspects of the logic model and its implementation. A logic model can be fit for purpose but if it is misunderstood or incorrectly implemented it is of little practical use. There could be stronger language around how ill-defined key components of the programme appeared to be for participants within your data. For example, the discrepancy as to what the intended impact of the programme is constitutes a huge problem. As you highlight in the introduction the development outcomes need to be prioritised within SfD and a lack of clarity around them is a huge flaw within programming. I understand the need for sensitivity with partner organisations, but this is the purpose of this study and without such clarity future aims around effectiveness evaluation cannot occur. These recommendations exist within Table 2 but the importance/strength of these recommendations is not always mirrored in the discussion. These can be strengthened while still celebrating P3P for their willingness to engage with independent researchers to explore their intervention. | Thanks also for this valuable comment. We added some explanations and a few sentences in the discussion section to address these points. See p.18, l.548-589. |

**REVIEWER 2**

**RESPONSES TO REVIEWERS**

| **COMMENTS** | **RESPONSES TO REVIEWER 2** |
| --- | --- |
| This is a well written and organised paper, with a coherent narrative and argument, and which moves towards enhancing our knowledge of the use of logic models in the Sport for Development field. | Thanks for your comments, there are very appreciated! You perfectly understand what is the aim of this study and what we intended to do. |
| I have only a few suggested issues for the authors to consider:  - There could be a more rigorous discussion of the role of logic models in both the realm of sport (not just SfD) and the realm of social intervention (social work, community work, development work). SfD sits at the intersection of these two realms, so there needs to be some consideration of where logic models fit in, in these respective fields. | We added a few sentences on this point. See p.19-20, l.548-589. |
| - Some justification of the focus on logic models compared to ‘theories of change’ should be given. The latter tend to be more prominent in SfD agencies and programs, so why focus on the former here? | We added some elements to explain this point. See p.4 l.101-112 in the introduction. |
| - I’m surprised that there is not more discussion of Coalter’s work with respect to the critical evaluation of SfD programs. Coalter’s recent work on program theory may be particularly salient here. | Thanks for your comment, we tried to rearrange this part following your proposition.  We introduced his works (p.3, l.74-76, l.105) and we discussed Coalter’s work in the discussion section, quoting your recommendation (p.18, l.548-564).  We consider your suggestion regarding Coalter’s recent work (Fred Coalter, Marc Theeboom & Jasper Truyens (2020) Developing a programme theory for sport and employability programmes for NEETs, International Journal of Sport Policy and Politics, 12:4, 679-697, DOI:10.1080/19406940.2020.1832136) on the program theory not perfectly fitting the content of our manuscript. We are already citing references quotes by Coalter (2020): Weiss 1995, 1998 and Pawson and Tilley 2004. |
| - The paper could and should respond more adequately in the conclusion to the routine ‘so what?’ question. There should be greater consideration of what these findings may mean for future research and studies, as well as practice, in the SfD field, and also, perhaps more importantly, for researchers in broader fields in sport or community/social development or youth work. These latter points are especially relevant given the audience of the journal, which is far wider than the sports studies field. | Thanks for your comment, we tried to rearrange this part following your proposition. See p.19-20 et l.574-589. |
