# Supplementary material for: Analyzing a sport for development program’s logic model by using key actors’ perceptions: The case of Pour 3 Points organization in Montreal
Source: PLoS One. 2022 May 12;17(5):e0267785. doi: 10.1371/journal.pone.0267785 (PMC9098001; doi:10.1371/journal.pone.0267785)
Supplement: Attachment — Submitted filename: Responses to reviewers PlosOne P3P round 2 TG 23 03 2022.docx [file pone.0267785.s003.docx]

**PONE-D-21-03672**

**Analyzing a SDP program’s logic model with key actors’ perceptions The case of Pour 3 Points organization in Montreal.**

**PLOS ONE**

**REVIEWER 1**

| **COMMENTS** | **RESPONSES TO REVIEWERS** |
| --- | --- |
| Reviewer #1: I thank the authors for their clear responses to previous comments. See below for remaining minor recommendations. | Thanks for your kind comments. |
| Language  Review language throughout, some long sentences could do with breaking up for clarity. Specific lines highlighted for review of sense/clarity:  Line 86 review use of term ‘real’ what is meant by this  Line 90-91 starting 'This literature review,' review for clarity  Line 108-110 starting 'Ridde and Dagenais,' review for clarity  Line 568-570 starting 'It could be considered,' review for clarity  Line 587-589 starting 'In reality, those,' review for clarity | We made the changes  l.78 we erased “real”  l.82 we erased “review”  l.100 we keep the sentences as it stands because we mentioned authors point of view.  l.558 we keep the sentences as it stands. This is exactly the idea we want to focus on.  l.577 we erased “in reality” |
| Throughout paper remove use of 1st person (‘we’, ‘our’). Currently mixes 1st and 3rd person references to authors. Consistent use of 3rd person (such as ‘the authors’ or ‘the research team’) preferred. | We made all the changes |
| Content  Line 221 clarify if interviews totalled 60mins or were on average 60mins each | We made the clarification |
| Line 254, include your definition of bracketing as it has been utilised in varied manners across different forms of Qual research. | l. 247 We included our definition as you suggested |
| Line 259 onwards, good inclusion of positionality and background. A line about different contributors’ backgrounds professionally (relevant professional experiences and paradigms of interest) would further strengthen this section. | L. 255 We added some elements regarding your comment. |
| Language around impact much better but would suggest a final check on all language to ensure nothing could read as having been objectively measured. Example line 640 Findings reveal also that the program is successful for all key actors should be changed to Findings reveal also that the program is perceived as successful for all key actors. Such simple changes prevent any misinterpretation of impact findings. | Thanks for this relevant comment. We did a deep modification of those aspects inside the text. |
